# Accumulation of prelamin A compromises NF-κB-regulated B-lymphopoiesis in a progeria mouse model

**DOI:** 10.1186/2046-2395-2-1

**Published:** 2013-01-02

**Authors:** Baohua Liu, Shuangcheng Zhou, Xinguang Liu, Keyuan Zhou, Fengju Zhang, Zhongjun Zhou

**Affiliations:** 1 Department of Biochemistry, Li Ka Shing Faculty of Medicine, The University of Hong Kong, 21 Sassoon Road, Pokfulam, Hong Kong; 2 Guangdong Medical College, 1 Xin Cheng Avenue, Dongguan, 523808, China; 3 Eye Center, Beijing Tongren Hospital, Capital Medical University, Beijing, 100730, China

**Keywords:** Aging, Hutchinson-Gilford progeria syndrome (HGPS), Zmpste24, Lamin A, B-lymphopoiesis, NF-κB

## Abstract

**Background:**

Alteration in the immune system is one of the most profound aspects of aging. Progressive changes in the number of B lymphocyte progenitors during aging have been reported but the underlying mechanisms are still elusive. A heterozygous G608G mutation in the *LMNA* gene leads to a deletion of 50 amino acids in lamin A protein, termed progerin, and is the predominant cause of Hutchinson-Gilford progeria syndrome (HGPS). Lack of Zmpste24, a metalloproteinase responsible for prelamin A processing, leads to progeroid features resembling HGPS. Therefore Zmpste24-deficient mice provide an ideal mouse model to study the impact of lamin A and (premature) aging on the aging-related decline of B lymphopoiesis.

**Results:**

Analysis of bone marrow (BM) nucleated cells revealed a decline of early B cell progenitors in *Zmpste24*^−/−^ mice. BM transplantation in a congenic strain completely rescued the defects in B lymphopoiesis, indicating that the decline in B cell progenitors in *Zmpste24*^−/−^ mice is attributable to defective BM microenvironments rather than to cell-intrinsic defects. Further investigation revealed downregulation of a set of important early B lymphopoiesis factors in *Zmpste24*^−/−^ bone marrow stromal cells (BMSCs), such as Vcam-1, SDF-1α, Flt3L and TSLP, and most of them are under transcriptional control of NF-κB signaling. Though TNFα stimulates IκBα degradation and NF-κB nuclear translocation in *Zmpste24*^−/−^ BMSCs, NF-κB fails to stimulate IκBα re-expression, which mediates a negative feedback loop of NF-κB signaling in wild-type BMSCs.

**Conclusions:**

Our data demonstrate a cell-extrinsic defect of B cell development in a progeroid mouse model and a critical role for lamin A in the regulation of NF-κB signaling and cytokines that are essential for lymphopoiesis.

## Background

Aging is a progressive deterioration of physiological functions that are necessary for survival and fertility [1]. Alteration in the immune system is one of the most profound aspects of aging, including shrinkage of the diverse repertoire of immunoglobins in both B and T lymphocytes [2,3], compromised immune responsiveness to pathogens, which is marked by greater proportion of low-affinity antibodies and involves both humoral and cell-mediated immune response [4,5], and increased auto-reactive cells leading to higher risk of autoimmune diseases [6].

B lymphocytes are one of the main components of the adaptive immune system and are responsible for the generation of B cell receptors (BCRs, also known as immunoglobulins), which recognize a large repertoire of antigens [7]. The B cell development is a highly ordered process orchestrated by differentiation from hematopoietic stem cells (HSCs). The initial commitment to the B cell lineage is characterized by the expression of CD45R/B220, leading to the earliest fraction of B cell progenitors, precursor of B cell progenitor (pre-pro-B) [8,9]. Pre-pro-B cells give rise to progenitor B (pro-B) cells [10,11]. The following stage is B cell precursors (pre-B), comprising mainly small resting cells. The subsequent expression of surface immunoglobulin M (sIgM) is the hallmark of the progression from pre-B cells to immature B cells when they start to leave bone marrow (BM) niches and enter the peripheral blood for further maturation. Contrasting to myeloid compartments, which are relatively intact during aging, B lymphopoiesis declines significantly with age [12]. However, the underlying mechanisms remain elusive.

Hutchinson-Gilford progeria syndrome (HGPS) is an extremely rare genetic disorder of early onset premature aging. Patients with HGPS can only live for 12 to 16 years and are clinically characterized with early growth retardation, small body size, lipodystrophy, loss of hair, stiff joints, reduced bone density, dilated cardiomyopathy and atherosclerosis [13,14]. HGPS is predominantly caused by a *de novo* p.G608G lamin A mutation. Lamin A is first synthesized as prelamin A with an additional 18 amino acids on the C-terminus, which dictates a series of processing events involving farnesylation, proteolysis and methylation [15-17]. ZMPSTE24, a metalloprotease, is required for the proteolytic cleavages during lamin A maturation [18]. The G608G mutation activates a cryptic splicing donor signal in exon 11, leading to a 150-nucleotide deletion in the *LMNA* transcript and a 50-residue truncation in the prelamin A protein, referred to as progerin. Progerin lacks the second proteolytic cleavage site of ZMPSTE24 but retains the CAAX motif [19,20]. Mice lacking *Lmna* surfer from growth retardation and muscle dystrophy, resembling Emery-Dreifuss muscular dystrophy (EDMD) [21]; depleting Zmpste24 in mice recapitulates many progeroid features found in HGPS patients [16]. *Lmna*^−/−^*Zmpste24*^−/−^ double knockout mice phenotypically resemble *Lmna* single knockouts, while depleting only one allele of *Lmna* ameliorates progeroid phenotypes and extends lifespan in *Zmpste24*^−/−^ mice [22,23]. This suggests that prelamin A is most likely the only substrate of Zmpste24, and unprocessed prelamin A is the direct cause of premature aging imposed by Zmpste24 deficiency.

Alternate splicing also happens at the wild type *LMNA* locus, leading to expression of low levels of progerin [24] and indicating that HGPS might share, at least partially, mechanism(s) with normal aging process. This idea is supported because the expression of ectopic progerin results in defective proliferation and premature senescence in human cells [25,26]; the number of progerin positive cells gradually increase during aging in normal individuals [27]; and telomere shortening or dysfunction activates progerin production [28]. Therefore Zmpste24-deficient mice provide an ideal mouse model to study the impact of lamin A and (premature) aging on the decline of B lymphopoiesis. It has been recently reported that loss of *Lmna* in mice causes non-cell autonomous defects of B cell development, possibly attributable to compromised bone marrow stromal cells (BMSCs) and/or the overall unhealthiness of the mutants [29]. In the current study, we asked whether the accumulation of prelamin A affects B lymphopoiesis in *Zmpste24*^−/−^ mice. We found an extrinsic decline of early B lymphocyte progenitors in *Zmpste24*^−/−^ mice. Defects in early B lymphopoiesis are most likely attributable to defective BM niches as *in vitro* cultured *Zmpste24*^−/−^ BMSCs are compromised in NF-κB signaling and the secretion of cytokines necessary for early B lymphopoiesis, including Vcam-1, SDF-1α, Flt3L, TSLP, *etcetera.* Our data reveal a critical role for lamin A in regulating NF-κB signaling that is essential for lymphopoiesis.

## Results

### Defective B lymphopoiesis in *Zmpste24*^−/−^ mice

**Figure 1:**
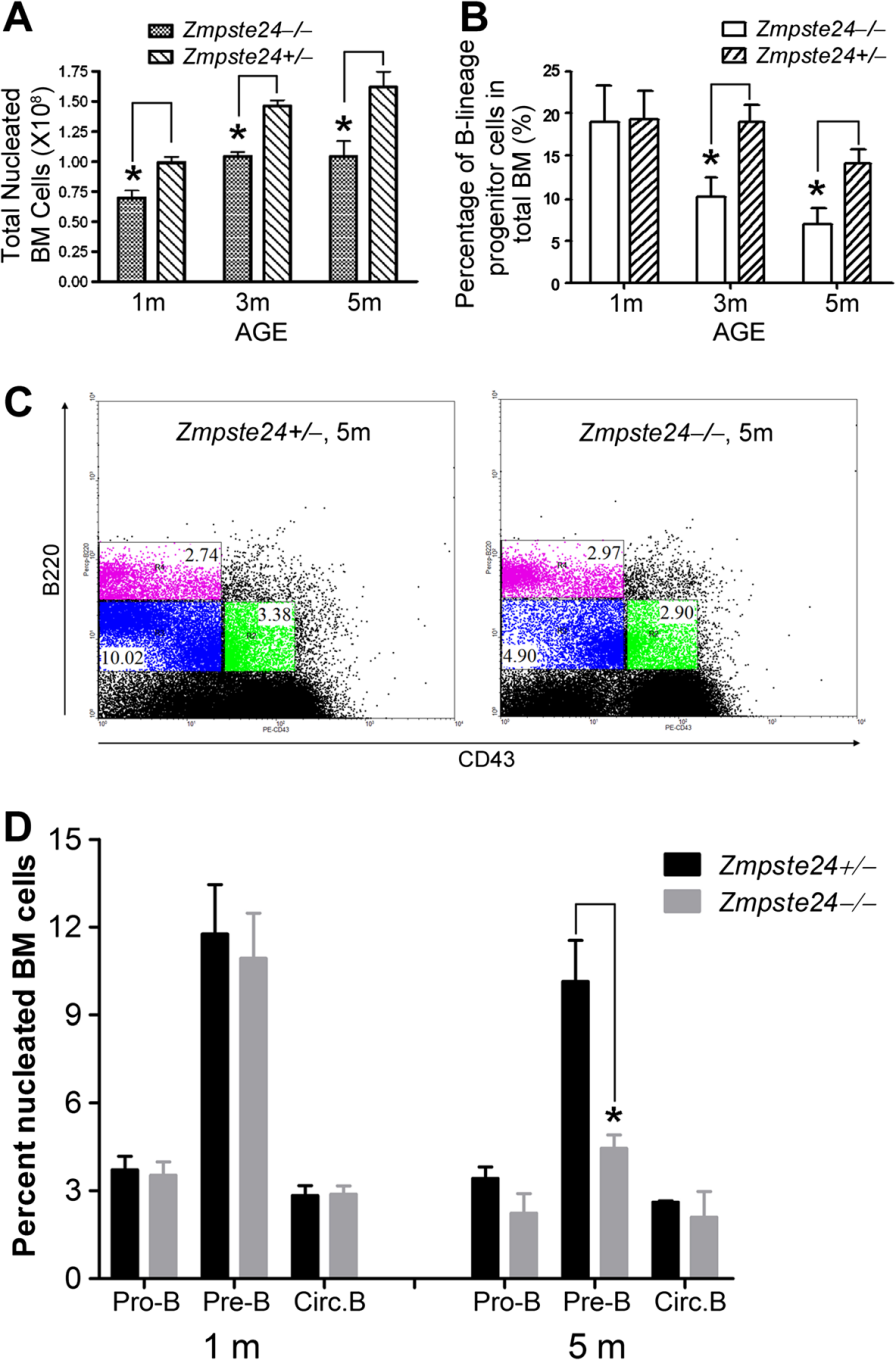
**Defective B cell development in *Zmpste24***^**−/− **^**mice.** (**A**) Decreased bone marrow nucleated cells in *Zmpste24*^−/−^ mice compared to their wild-type littermates. Data represent mean ± SEM, n ≥5. *P <0.05. (**B**) Decreased B lymphocytes in *Zmpste24*^−/−^ mice started from 3 months of age, compared to their wild-type littermates. Data represent mean ± SEM, n = 5. *P <0.05. (**C**) Representative B cell profile in 5-month-old *Zmpste24*^−/−^ mice and control, showing decreased pro-B (B220^lo^CD43^+^) and pre-B (B220^lo^CD43^−^) cells. (**D**) Decreased pro-B and pre-B cells in 5-month-old *Zmpste24*^−/−^ mice. Data represent mean ± SEM, n = 5. *P <0.05. circ. B, circulating B cells; pre-B cells, B cell precursors; pro-B cells, progenitor B cells.

We first examined whether there is any difference in the B cell development in *Zmpste24*^−/−^ mice. As shown in Figure 1A, the number of nucleated BM cells from femurs and tibias decreased in *Zmpste24*^−*/*−^ mice when comparing with *Zmpste24*^+/−^ mice, which are phenotypically indistinguishable from their *Zmpste24*^+/+^ littermates. Of note, while the number of nucleated BM cells increased along with age in *Zmpste24*^+/−^ mice, no further increase was observed after 3 months of age in *Zmpste24*^−/−^ mice. We then performed FACS analysis on *Zmpste24*^−/−^ mice at 1 month, 3 months and 5 months of age using their sex-matched heterozygous littermates as controls. While 1-month-old *Zmpste24*^−/−^ mice had a similar profile of early B cells to that of their heterozygous littermates, *Zmpste24*^−/−^ mice of 3 months and 5 months of age showed a significant decrease in the number of B lymphocytes as well as the percentage of pro-B (identified as B220^lo^CD43^+^) and pre-B (identified as B220^lo^CD43^−^) populations (Figure 1B-D), suggesting a defect in the generation of B lineage lymphocyte progenitors, which is consistent with the previous reports on physiologically senescent mice [30-32].

### Defective B lymphopoiesis in *Zmpste24*^−/−^ mice is not cell-intrinsic

**Figure 2:**
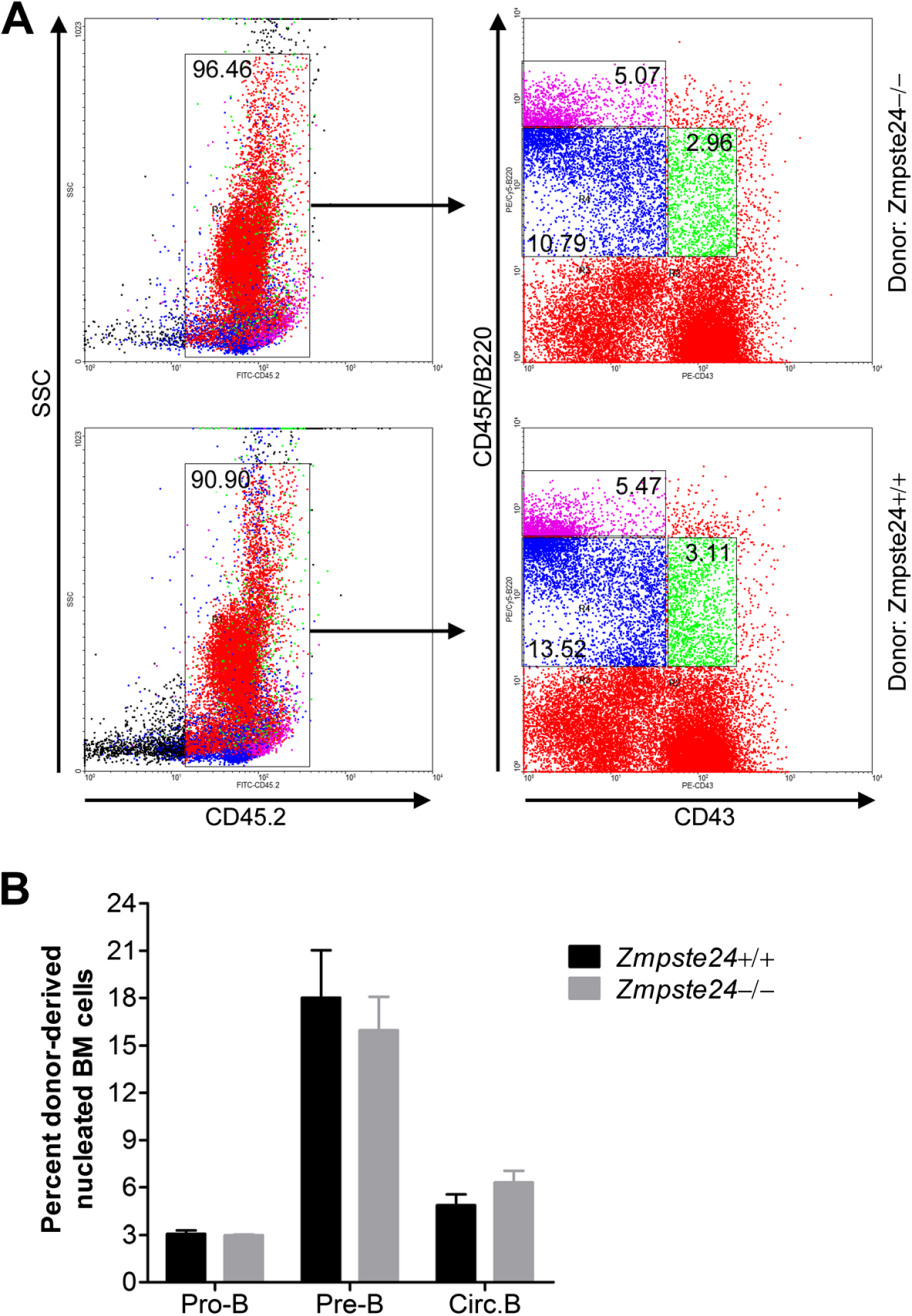
**Defective early B lymphopoiesis in *Zmpste24***^**−/− **^**mice is cell-extrinsic.** (**A**) Representative plots showing B cell profile (pro- and pre-B) analyses in recipients with *Zmpste24*^−/−^ or *Zmpste24*^+/+^ donor cells. (**B**) Detailed B cell profile analyses, including the number of pro-B cells, pre-B cells and circ. B cells, showed a complete rescue of defective early B cell development in *Zmpste24*^−/−^ mice. Data represent mean ± SEM, n = 4. circ. B, circulating B cells; pre-B cells, B cell precursors; pro-B cells, progenitor B cells.

The defective B lymphopoiesis observed in *Zmpste24*^−/−^ mice could be either cell-intrinsic or cell-extrinsic. To identify this, the BM transplantation test was performed with congenic strain B6 as recipients. A comparable number of nucleated BM cells from either 6-month-old *Zmpste24* null mice or their wild-type littermates was transplanted into lethally irradiated B6 mice and kept for 2 months before fluorescence-activated cell sorting (FACS) analysis. The BM cells of donor origin (CD45.2^+^) are identified with FITC-CD45.2 antibody. Two months after the transplantation, FACS analysis showed that the engraftment efficiency is between 90% and 96% (Figure 2A). However, the donor-derived cells of *Zmpste24* null mice showed no significant difference in the profiling of early B cell development, including the proportions of B lineage progenitors and circulating B cells when compared with wild-type origin (Figure 2). These results indicate that the defects observed in early B cell development in *Zmpste24-*deficient mice are not cell-intrinsic, but rather the consequence of a defective BM microenvironment.

### Compromised bone marrow niches in *Zmpste24*^−/−^ mice

**Figure 3:**
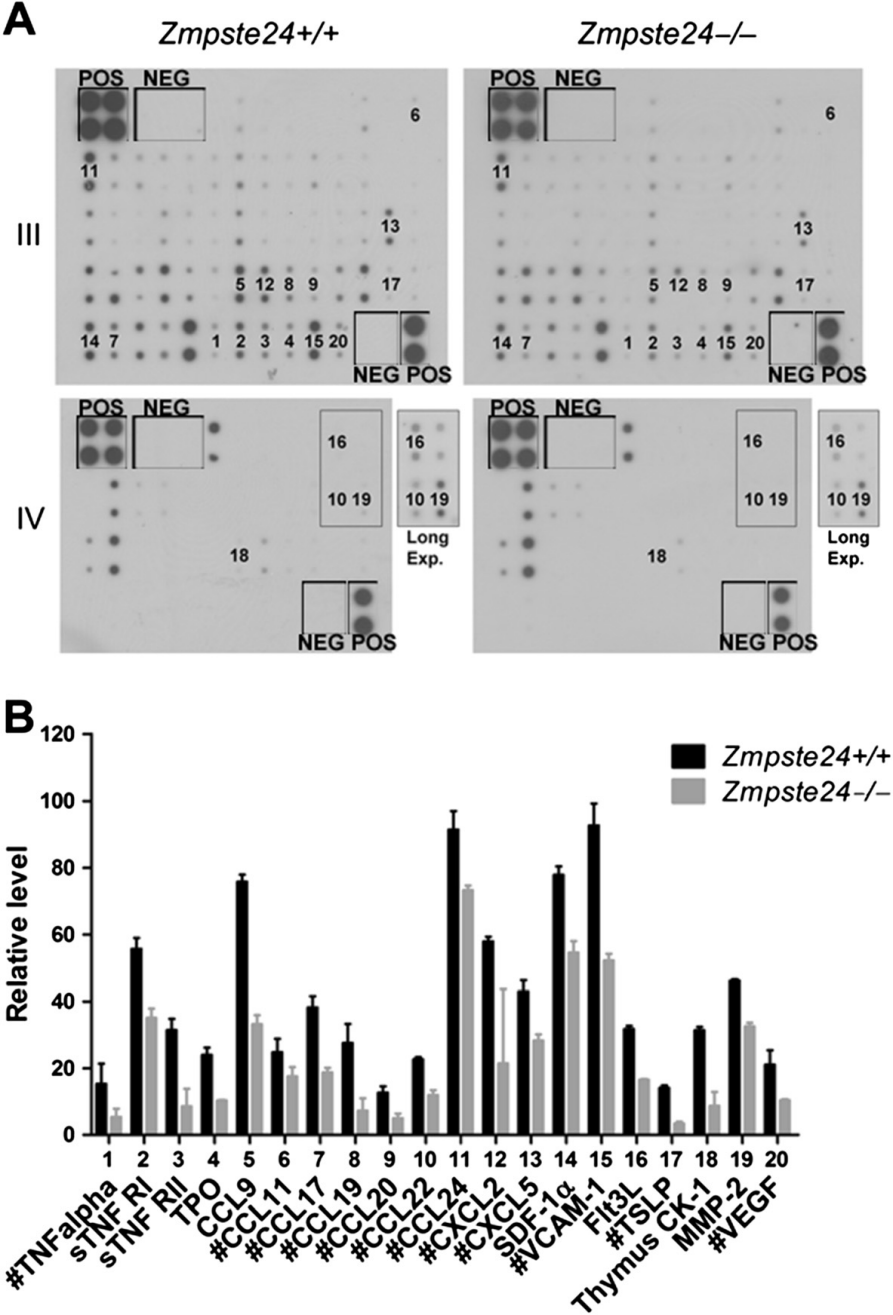
**Reduced essential factors required for B lymphopoiesis owing to defective NF-κB signaling in *Zmpste24***^**−/− **^**bone marrow stromal cells. **(**A**) Representative blots showing cytokine expression in wild-type and *Zmpste24*^−/−^ bone marrow stromal cells (BMSCs). Corresponding cytokines showed significant difference in (**B**) were labeled with number. (**B**) Downregulated cytokines in *Zmpste24*^−/−^ BMSCs and most of them (#) are under transcriptional control of NF-κB, especially those required for early B cell development, including Vcam-1, SDF-1α, Flt3L and TSLP. Data represent mean ± SEM, n = 3. *P* <0.05, *Zmpste24*^−/−^ BMSCs *versus* wild-type. POS, positive control (GAPDH); NEG, negative control (Blank).

The bone marrow hematopoietic niches provide cytokines and cell adhesion molecules essential for B cell development and survival [10,33]. However the cellular components in bone marrow niches are highly complex and still poorly understood. Recent studies revealed that a subset of bone marrow stromal cells (BMSCs) expressing vascular cell adhesion molecule-1 (Vcam-1) is essential for B-lymphopoiesis [34]. Of these reticular Vcam-1^+^ stromal cells, roughly 46% express interleukin 7 (IL-7) and around 17% express stromal cell-derived factor-1α (SDF-1α), also known as chemokine (C-X-C motif) ligand 12 (CXCL12) or pre-B-cell growth-stimulating factor (PBSF), both of which are critical for B lymphopoiesis [35]. To investigate the potential niche defects responsible for the impaired B lymphopoiesis in *Zmpste24*^−/−^ mice, BMSCs were cultured and subjected to cytokine array from 6-month-old *Zmpste24*^−/−^ mice in comparison with their heterozygous littermates. With quantification by Image J, 20 out of 96 cytokines showed significant alteration in their expression level (Figure 3 and [see Figure S1 in Additional file 1) and most of them are transcriptional targets of NF-κB ([See Figure S2 in Additional file 2 and online source at http://www.bu.edu/nf-kb/gene-resources/target-genes/), indicating a defective NF-κB pathway in *Zmpste24*^−/−^ mice. Among those affected, Vcam-1, SDF-1α, tyrosine kinase 3 ligand (Flt3L) and thymic stromal lymphopoietin (TSLP) are most important for the early B cell development; Vcam-1, SDF-1α and TSLP are under transcriptional control of NF-κB signaling [36-38].

### Defective NF-κB pathway in *Zmpste24*^−/−^ mice

**Figure 4:**
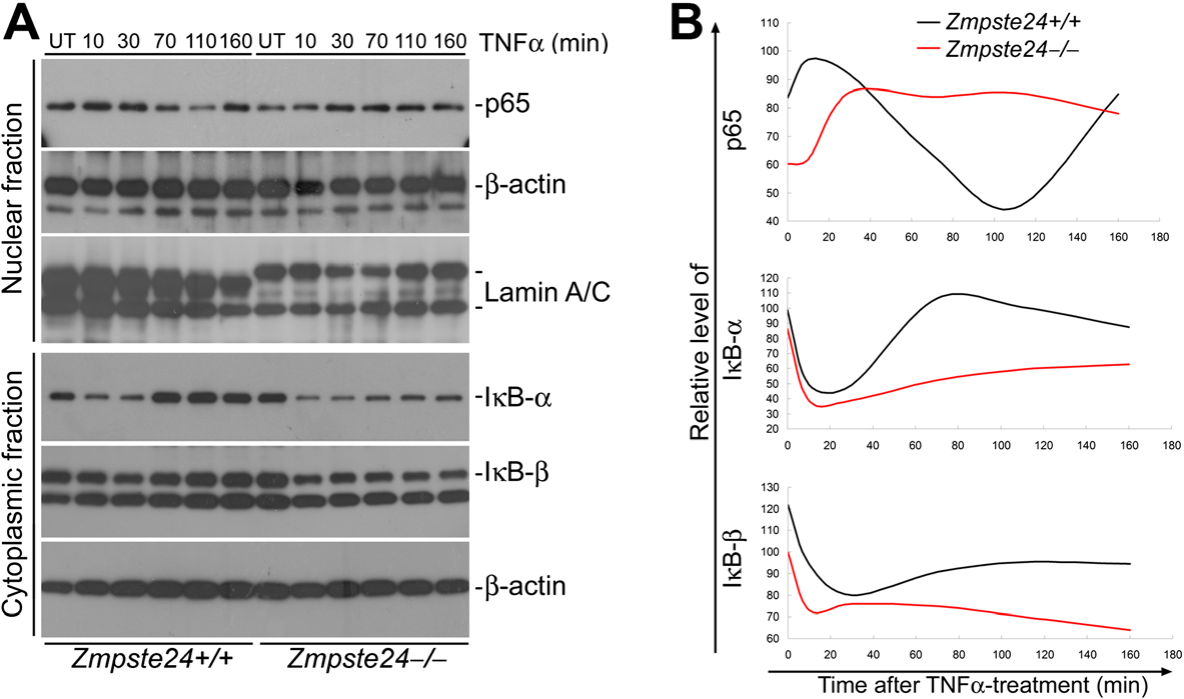
**Defective NF-κB signaling in *Zmpste24***^**−/− **^**bone marrow stromal cells. **(**A**) Representative western blotting results showing that upon transient TNFα stimulation, NF-κB was defective in activating IκBα/β expression and the following negative feedback in Z*mpste24*^−/−^ bone marrow stromal cells (BMSCs). (**B**) Quantification of p65 and IκBα/β after TNFα treatment in (**A**) by Image J (see Materials and Methods)

In canonical NF-κB pathway, the p65/p50 complex is sequestered in cytoplasm by IκBα/β/ε; upon cytokine stimulation (for instance, TNFα), IκBα/β/ε is phosphorylated by IκB kinase β (IKKβ) and subjected to ubiquitination and proteasomal degradation, and then the p65/p50 complex is released and enters the nucleus to activate the relative gene expression [39]. NF-κB pathway activation is tightly regulated by an IκB-NF-κB negative feedback loop [40]. In Hoffmann’s model, the nuclear p65/p50 complex activates transcription of IκBα/β/ε, and newly synthesized IκBα/β/ε inhibited amplification NF-κB signaling. Analyses of the affected chemokines and TNFα family members by STRING 8.2 (an online tool for analysis of protein-protein interaction) suggested a defect in NF-κB pathway in *Zmpste24*^−/−^ BMSCs [See Figure S2 in Additional file 2. As determined by nuclear fractionation and western blotting, compared with wild-type, a downregulation of the nuclear level of p65 in *Zmpste24*^−/−^ BMSCs was found (Figure 4, untreated). We further investigated the dynamic changes of NF-κB signaling in *Zmpste24*^−/−^ and wild-type BMSCs upon stress. We transiently stimulated BMSCs with TNFα and examined the nuclear translocation of p65 protein and cytoplasmic level of IκBα/β. As shown in Figure 4, in *Zmpste24*^*+*/+^ BMSCs, the nuclear level of p65 peaked at 10 minutes, then gradually decreased to the lowest level until 110 minutes after the stimulation and finally stabilized to normal level (before treatment) around 160 minutes after the stimulation. Tightly correlated with the dynamic changes of p65 level, the lowest level of IκBα/β was observed around 10 minutes after stimulation and went to the highest level at 110 minutes and stabilized around 160 minutes. These results show a beautiful negative feedback loop of IκB-NF-κB pathway. However, in *Zmpste24*^−/−^ BMSCs, the highest level of nuclear p65 was slightly delayed until 20 minutes and then remained at the highest level until 160 minutes. In consistent with the changes of p65, IκBα/β went to its lowest level around 20 minutes after stimulation and remained unchanged until 160 minutes. Since IκBα/β is transcriptionally activated by NF-κB, these data suggested that although the nuclear accumulation of p65 was not much affect, the transcriptional activity of NF-κB was significantly compromised in *Zmpste24*^−/−^ BMSCs.

## Discussion

Bone marrow (BM) hematopoietic niches are essential for B cell development; they provide cytokines and cell adhesion molecules that are necessary for the survival of the B cells [10]. About two decades ago, Whitlock and Witte showed that adherent non-hematopoietic cells have great potential in supporting B-lymphopoiesis [41]. However the cellular basis that regulates B-lymphopoiesis is still poorly understood. Recent studies found that a subset of bone marrow mesenchymal cells expressing Vcam-1 is critical for B-lymphopoiesis [34,35]. Schaumann *et al*. found that FACS-purified adherent Vcam-1^+^ stromal cells share similar surface markers with BMSCs, such as CD13, CD31, CD90, CD105, *etcetera*[34], suggesting that BMSCs, at least the Vcam-1^+^ subpopulation, might recapitulate part of the *in vivo* B cell niches. In the current study, we did cytokine array analysis for all BMSCs regardless of the expression of Vcam-1 because all BM-derived adherent non-hematopoietic cells have potentials to support B-lymphopoiesis [41]. We found that a series of important early B cell factors, including Vcam-1, SDF-1α and Flt3L, are primarily and significantly affected in *Zmpste24*^−/−^ BMSCs. Vcam-1 interacts with very late antigen integrins (VLA-4), thus mediating the adhesion of B cell precursors to stromal cells [42,43]. Depleting Vcam-1 in mice results in defective B cell homing to the bone marrow [44,45]. SDF-1α belongs to chemokine family and is mainly recognized by CXC-chemokine receptor 4 (Cxcr4). *Sdf1*^−/−^ embryos showed a significantly decreased number of the earliest stage of B cell precursors in fetal liver [46]. Adoptive transfer experiments showed that *Cxcr4*^−/−^ fetal liver cells failed to generate pro-B cells [47]. In chimeric mice reconstituted with *Cxcr4*^−/−^ fetal liver cells, the number of donor-derived pro-B and pre-B cells was significantly decreased in BM but increased in peripheral blood [48,49]. It has been shown that Vcam-1 is expressed in almost all of the SDF-1α-expressing cells, whereas only 17% of all the Vcam-1^+^ stromal cells are SDF-1α positive and the rest comprises of a high proportion of IL-7^+^ cells [34,35]. IL-7 is the first defined cytokine essential for B cell development [50]. Research based on both gene targeting and *in vitro* culture have shown that IL-7 is necessary for the development from pre-pro-B and pro-B stages [10,51]. Although no significant change of IL-7 expression was observed in *Zmpste24*^−/−^ BMSCs (data not shown), we found that Flt3L, which synergizes with IL-7 to support early B cell development [52], is significantly decreased. Therefore defects in both Vcam-1^+^SDF-1α^+^IL-7^−^ and Vcam-1^+^SDF-1α^−^IL-7^+^ bone marrow stromal cells might be responsible for the defective B-lymphopoiesis in *Zmpste24*^−/−^ mice. In addition to Vcam-1, SDF-1α and Flt3L, 17 other cytokines also show significant changes in *Zmpste24*^−/−^ BMSCs (see Figure 3). Some of these cytokines, for example, TSLP, are important for early murine B cell development [53]. However it is still unclear whether changes in these cytokines represent defects in Vcam-1^−^ BMSCs that might also be responsible for the defective B-lymphopoiesis in *Zmpste24*^−/−^ mice. Our data are consistent with a previous report showing extrinsic defects in B and T cell development in *Lmna* null mice [29]. However, Hale and colleagues showed that engrafted *Lmna* null thymus is comparable in the ability to support T lymphopoiesis and concluded that the defective T cell development is attributable to the overall unhealthiness of the host animal instead of impaired stromal cells. In the current study, we employed *in vitro* expanded BMSCs; therefore, the defects in various cytokines are independent of the overall healthiness of examined animals. In this regard, the effects of lacking lamin A in *Lmna* null mice and accumulation of prelamin A in *Zmpste24* null mice are likely different in the regulation of lymphopoietic niches.

Our data are also consistent with a report showing defective NF-κB pathway in *Lmna*^−/−^ mice [54], where loss of *Lmna* compromised IL-1β-stimulated NF-κB-regulated luciferase activity, although the binding of NF-κB to target sequences was increased. It is worthwhile to elucidate the underlying molecular mechanism of defective NF-κB signaling in *Zmpste24*^−/−^ mice in future studies. The transcriptional activity of NF-κB can also be regulated by co-activators, including p300/CBP, PCAF, p160 proteins (SRC-1/2/3), *etcetera*, and co-repressors, such as HDAC1/2/3, SMRT, NcoR, *etcetera*, which modulate local chromatin structure and NF-κB signaling. Given that the chromatin structure is disorganized in HGPS and normal aging cells [24,55,56], one possibility is that accumulation of unprocessed prelamin A may affect local chromatin remodeling and thus impede NF-κB-mediated transcription activation in *Zmpste24*^−/−^ BMSCs. This may also explain the finding that only a set of targets are affected by defective NF-κB signaling in *Zmpste24*^−/−^ BMSCs.

## Conclusions

In this study, we found a significant decline in the number of B cell progenitors in *Zmpste24*^−/−^ mice. Further investigation revealed that the defective B-lymphopoiesis is most likely attributable to decreased NF-κB signaling in BMSCs, which likely resembles *in vivo* B cell niches. Of those 20 affected cytokines in *Zmpste24*^−/−^ BMSCs, Vcam-1, SDF-1α, Flt3L and TSLP are among the most well-studied and are essential for early B lymphopoiesis. Collectively, our data demonstrate a cell-extrinsic defect of B cell development in a progeroid mouse model and a critical role for lamin A in the regulation of NF-κB signaling and essential cytokines in B-lymphopoiesis. As progerin accumulates in and contributes to healthy aging, our data also suggest a mechanistic explanation for aging-related decline in B cell populations in aged individuals.

## Methods

### Antibodies

PE/Cy5 anti CD45R/B220 (RA3-6B2), R-PE anti CD43 and FITC anti-mouse CD45.2 were sourced from BioLegend (San Diego, CA, USA). Mouse lineage panel (anti-CD3ε, anti-CD11b, anti-B220, anti-Ly-6G, anti-Ly-6C, and anti-TER-119) and anti Biotin microbeads were purchased from BD Biosciences (San Jose, CA, USA). Rabbit anti p65, IκB-α, IκB-β and lamin A/C antibodies were from Santa Cruz (Santa Cruz, CA, USA). Mouse anti β-actin antibody was from Sigma (St. Louis, MO, USA).

### Animals

Mouse experiments were performed under the regulations and guidelines of the Committee on the Use of Live Animals in Teaching and Research (CULATR) at the University of Hong Kong. *Zmpste24*^−/−^ mice were described previously [16].

### B cell profile analysis and bone marrow transplantation

Bone marrow cells were flushed out into HBSS supplemented with 2% FBS, stained with B cell markers (PE/Cy5-anti-CD45R/B220 and R-PE-anti-CD43) and subjected to FACS analysis. For total bone marrow transplantation, 6-month-old B6.SJL/BoyJ mice were irradiated with 9 Gy and served as recipients. A total of 1×10^7^ bone marrow nucleated cells from either *Zmpste24*^−/−^ mice or wild-type littermates were suspended in 100 μl of HBSS supplemented with 2% FBS and injected into recipients via tail vain. FITC-anti-CD45.2 antibody was used to identify donor-derived lymphocytes.

### Bone marrow stromal cell culture and RayBio™ Mouse Cytokine Antibody Array

BMSCs were cultured as described [57]. Briefly, total bone marrow cells were flushed out with HBSS buffer (containing 2% FBS) and taken into culture with 3 ml α-MEM medium (containing 20% FBS and penicillin-streptomycin) directly in a 60-mm petri dish. Culture medium was replaced with fresh α-MEM (containing 20% FBS and no antibiotics) after 24 h, which cleared most of the unattached cells (including red blood cells) and cell debris. After 6 days, the P0 culture was trypsinized (with 0.025% trypsin and 0.01% EDTA) for 10 minutes and the detached cells were transferred into a 10-cm petri dish for culture. The cells were then subcultured every 3 to 4 days. After passage four, the BMSC cultures were subjected to magnetic sorting with BD mouse lineage panel (anti-CD3ε, anti-CD11b, anti-B220, anti-Ly-6G, anti-Ly-6C, and anti-TER-119) and anti Biotin microbeads according to the manufacture’s instruction. Cells from three different mice were pooled together for the sake of decrease the background noise and increasing the robustness of the difference between mutant and heterozygous mice in the following cytokine array and protein expression analysis. RayBio™ Mouse Cytokine Antibody Array III and IV (RayBiotech, USA) were used to compare the cytokine expression pattern in *Zmpste24*^−/−^ and *Zmpste24*^+/+^ BMSCs. The experiment was performed according to the instruction of the manufacturer.

### Protein extraction and western blotting

Total cell lysate was prepared by suspending the cells in five volumes of suspension buffer, and then adding five volumes of Laemmli buffer and boiling for 5 minutes. For protein fractionation, cells were suspended in 100 μl ice-cold buffer A (10 mM HEPES, pH 7.9, 10 mM KCl, 1.5 mM MgCl_2_, 0.34 M sucrose, 10% glycerol, 1 mM DTT, protease inhibitors). After the addition of 0.1% Triton X-100, the cell suspension was mixed gently, incubated on ice for 5 minutes and centrifuged at 1300×g at 4°C for 4 minutes. The supernatant (S1) was transferred to a new tube and clarified by high-speed centrifugation (12000×g, 10 minutes, 4°C). The remaining nuclei pellet (P1) was washed once with 100 μl buffer A and then resuspended in 100 μl Laemmli buffer and boiled for 5 minutes. Western blotting was performed as described previously [58].

### Image quantification and statistics analysis

Photos were processed with Photoshop CS® (Adobe Systems Incorporated, San Jose, CA, USA) when necessary. The pixel intensity of western blotting and dot blotting was measured by Image J gel analysis function [59] and normalized to housekeeping controls. Student’s *t*-test was performed for statistical comparison.

## Abbreviations

BCRs: B cell receptors; BM: Bone marrow; BMSCs: Bone marrow stromal cells; FACS: Fluorescence-activated cell sorting; HGPS: Hutchinson-Gilford progeria syndrome; HSCs: Hematopoietic stem cells; PBSF: Pre-B-cell growth-stimulating factor; pre-B: B cell precursors; pre-pro-B: Precursor of B cell progenitor; pro-B: Progenitor B; sIgM: Surface immunoglobulin; TSLP: Thymic stromal lymphopoietin; Vcam-1: Vascular cell adhesion molecule-1.

## Competing interests

The authors declare that they have no competing interests.

## Authors’ contributions

BL and ZZ designed experiments. BL and SZ conducted experiments. BL, XL, KZ, FZ and ZZ analyzed the data. BL, SZ and ZZ wrote the manuscript. All authors discussed the results and commented on the manuscript. All authors read and approved the final manuscript.

## Supplementary Material

Additional file 1 **Figure S1.** Cytokines that were not affected in *Zmpste24*−/− BMSCs. Click here for file

Additional file 2 **Figure S2.** Downregulated cytokines in *Zmpste24*−/− BMSCs are correlated with NF-κB pathway. (A) Interacting network among NF-κB pathway and downregulated factors in Zmpste24−/− BMSCs was predicted by online tool STRING (http://string.embl.de/). (B) Interacting network among those downregulated factors that are essential for B cell development and NF-κB signaling, predicted by STRING. *Significantly downregulated cytokines in *Zmpste24*−/− BMSCs. Click here for file
