# Supplementary figures and images for: Accumulation of prelamin A compromises NF-κB-regulated B-lymphopoiesis in a progeria mouse model
Source: Longev Healthspan. 2013 Jan 2;2:1. doi: 10.1186/2046-2395-2-1 (PMC3922919; doi:10.1186/2046-2395-2-1)

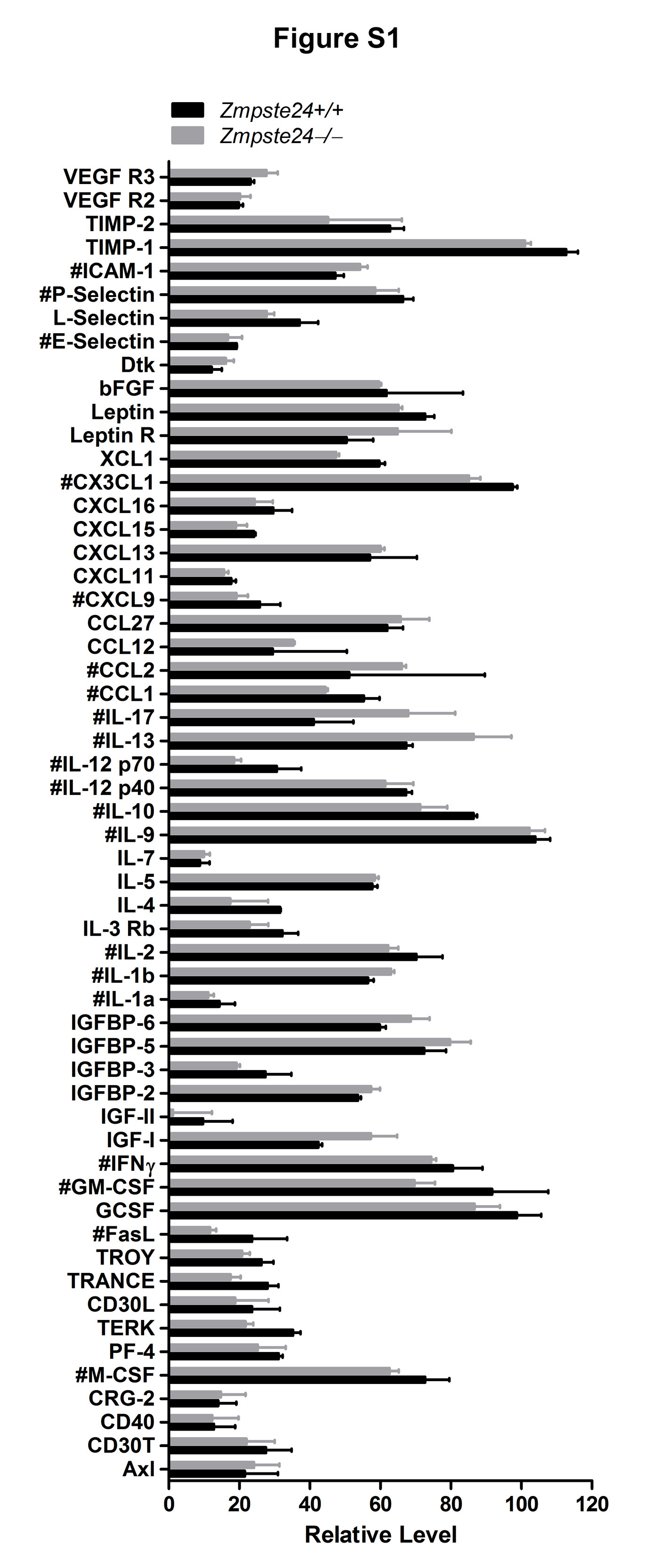

Supplement: Additional file 1 — Figure S1. Cytokines that were not affected in Zmpste24−/− BMSCs. [file 2046-2395-2-1-S1.jpeg]

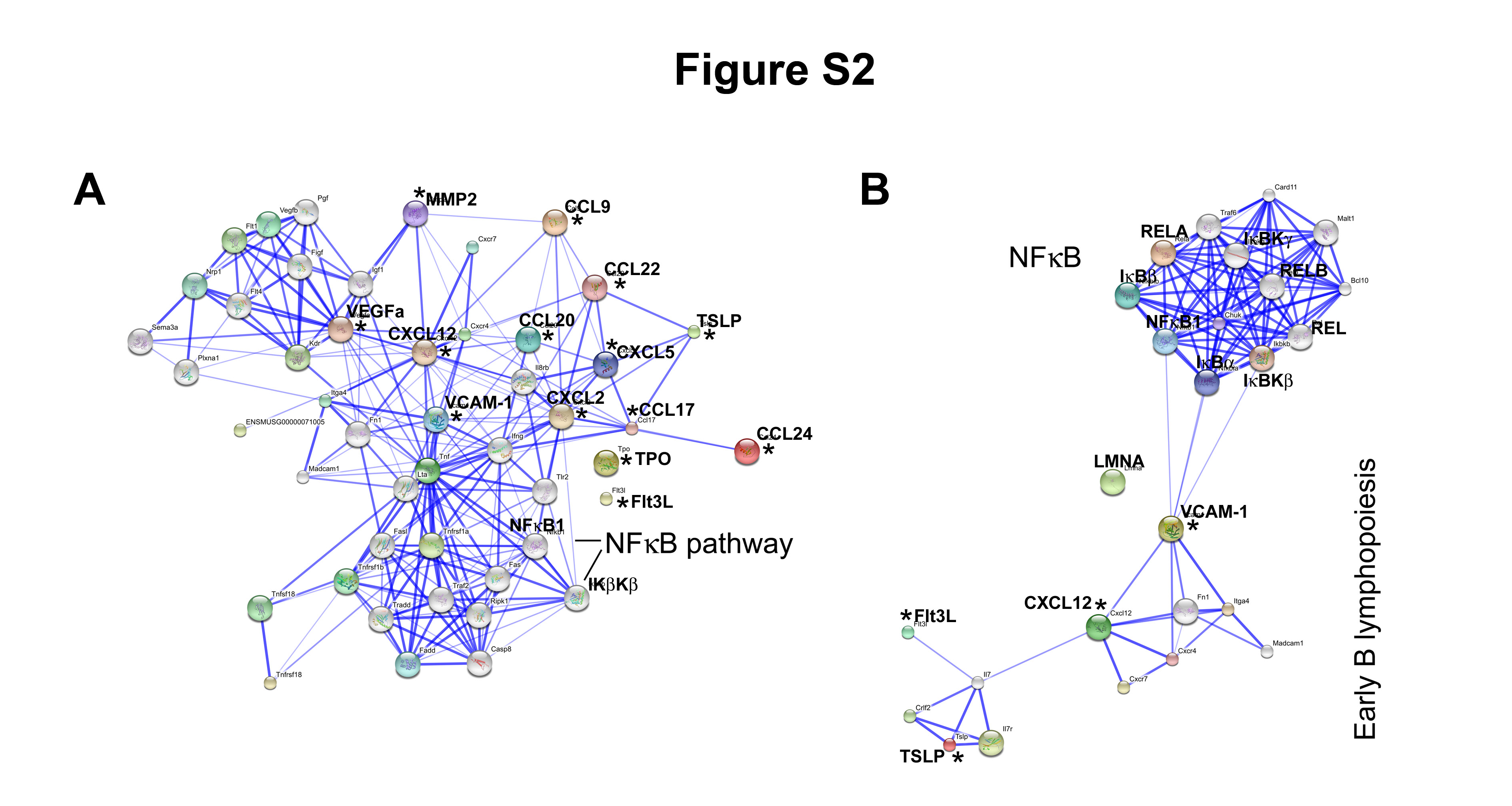

Supplement: Additional file 2 — Figure S2. Downregulated cytokines in Zmpste24−/− BMSCs are correlated with NF-κB pathway. (A) Interacting network among NF-κB pathway and downregulated factors in Zmpste24−/− BMSCs was predicted by online tool STRING (http://string.embl.de/). (B) Interacting network among those downregulated factors that are essential for B cell development and NF-κB signaling, predicted by STRING. *Significantly downregulated cytokines in Zmpste24−/− BMSCs. [file 2046-2395-2-1-S2.jpeg]
